# War-Related PTSD and Its Impact on Neuroendocrine Function, Immune Dysregulation, and Growth in Children: Implications for Syrian Children

**DOI:** 10.1055/s-0046-1824466

**Published:** 2026-06-17

**Authors:** Batool Sultan, Shriya Devendra Tayade, Riddhi Jadhav

**Affiliations:** 1 RAK Medical and Health Sciences University, Ras Al-Khaimah, United Arab Emirates

**Keywords:** Syrian children, refugees, war, posttraumatic stress disorder, HPA axis, cortisol, inflammation, growth

## Abstract

**Background/Objectives:**

War exposure and displacement increase the risk of posttraumatic stress disorder in children and may coincide with alterations in stress biology, inflammation, growth, and family functioning, with Syrian populations disproportionately affected.

**Methods:**

We conducted a targeted review informed by the SAMS 2025 poster bibliography with supplementary searches of PubMed/PMC and Google Scholar (2010–2025). Eligible studies included conflict-affected children/adolescents (0–18 years) with PTSD measures and at least one biological or developmental outcome. Two reviewers screened and extracted data and appraised study quality using JBI/NOS-aligned domains.

**Results:**

Evidence included school-based samples within Syria and Syrian refugee cohorts in host countries, alongside mechanistic reviews. Findings showed higher cortisol/HPA-axis activity reported in trauma-exposed pediatric samples, and Syrian refugee studies using hair cortisol suggest associations between war exposure and PTSD symptoms. Mechanistic literature suggests possible elevations in inflammatory markers (e.g., IL-6, CRP) in some contexts, but conflict-specific pediatric biomarker data remain limited. Growth impacts are plausibly mediated by cumulative adversity and nutrition insecurity, with sparse direct Syrian cohort evidence.

**Conclusions:**

Syrian conflict-affected children experience a substantial mental health burden, and available data suggest biological embedding of trauma via stress and immune pathways; however, most studies are observational and confounding limits causal inference. Epigenetic and intergenerational findings should be interpreted as preliminary and hypothesis-generating. Future longitudinal studies with standardized measures and careful confounding control are needed.

## Introduction

The Syrian conflict has created one of the largest displacement crises in modern history. Children are disproportionately affected, facing direct war exposure, loss of caregivers, disrupted education, and prolonged post-migration stressors. 1 Posttraumatic stress disorder (PTSD) is a common outcome in conflict-affected youth and often co-occurs with depression, anxiety, sleep disruption, and behavioral problems. Beyond mental health, chronic stress may become “biologically embedded” through neuroendocrine and immune pathways, with potential implications for growth, infection susceptibility, and longer term cardiometabolic risk. 2 The hypothalamic–pituitary–adrenal (HPA) axis is the body's core stress-response system, and cytokines (e.g., interleukin-6 [IL-6]) are immune signaling proteins that can reflect inflammatory activity. 2 3 4

The Syrian American Medical Society (SAMS) 2025 poster summarized emerging links between pediatric war-related PTSD and neuroendocrine, immune, and developmental outcomes. In response to editorial feedback, the present manuscript 5 avoids over-specific quantitative claims that are not consistently supported across studies, 6 explicitly acknowledges heterogeneity in HPA-axis findings, 7 distinguishes evidence from Syrian cohorts versus broader trauma literature, 3 and frames epigenetic and intergenerational mechanisms as preliminary and hypothesis-generating. 8

## Materials and Methods

### Review Design

We performed a targeted review of peer-reviewed human studies relevant to war-related PTSD in children, emphasizing Syrian cohorts where available. The approach was designed to support a clinically oriented synthesis rather than a formal meta-analysis, given variability in outcomes and measurement. 7

### Data Sources and Search Strategy

Seed articles were drawn from the SAMS 2025 poster bibliography and screened for relevance to pediatric war exposure and PTSD-related outcomes. We searched PubMed and PubMed Central (PMC) for studies published between January 1, 2010 and December 31, 2025 and screened Google Scholar for additional records and citation chaining. Search concepts combined population, exposure, and outcomes using: (child* OR adolescent* OR youth) AND (Syria* OR Syrian OR refugee* OR conflict OR war OR displacement) AND (post-traumatic stress OR PTSD OR trauma) AND (cortisol OR HPA OR hypothalamic OR cytokine* OR inflammation OR IL-6 OR CRP OR growth OR height-for-age OR development OR epigenetic*). We additionally hand-searched reference lists of included articles and relevant reviews.

### Eligibility Criteria

We included (1) original quantitative studies of children/adolescents (0–18 years) affected by armed conflict (within Syria or as Syrian refugees) that reported PTSD symptoms/diagnosis and at least one biological/developmental outcome (HPA-axis marker, immune/inflammatory marker, growth/anthropometry, or validated developmental outcomes), and (2) high-quality mechanistic reviews directly relevant to PTSD biology when pediatric Syrian data were limited. 3 We excluded studies not involving pediatric populations, not involving war-related exposure, or without outcomes relevant to the review domains. 9

### Study Selection and Data Extraction

Two reviewers independently screened titles and abstracts and then assessed full texts for inclusion; disagreements were resolved by discussion. Data extracted included setting, population, PTSD measurement approach, outcomes, study design, and main conclusions. 9 Due to heterogeneity in outcomes and measurement, we performed a qualitative synthesis rather than meta-analysis. 8

### Risk-of-Bias Assessment

Two reviewers assessed methodological quality at the study level. Cross-sectional and cohort observational studies were appraised using Joanna Briggs Institute (JBI) critical appraisal checklists, and cohort studies were additionally evaluated using domains aligned with the Newcastle–Ottawa Scale (NOS). We rated each study as low, moderate, or high risk of bias based on participant selection, exposure and outcome measurement validity, confounding control, and completeness of reporting. Risk-of-bias judgments were used to qualify the strength of inferences and to guide cautious interpretation of mechanistic and intergenerational claims. 8 10

## Results

### Overview of Included Studies

Included studies comprised cross-sectional school-based surveys conducted within Syria, refugee cohorts in host countries (Jordan, Lebanon, Turkey, Greece, and Iraq), longitudinal cohorts evaluating hair cortisol as an index of HPA-axis activity, and mechanistic reviews describing immune and intergenerational pathways relevant to PTSD ( Table 1 ) 2 4 10 11

**Table 1:** The characteristics, PTSD measures, and principal findings of the included studies and reviews 1 2 3 4 5 6 7 8 9 10 11 12 13 14 15 16

Study	Population/setting	PTSD measure	Key findings relevant to this review (qualitative)
Perkins et al (2018) 5	492 school children (8–15 years), Damascus and Latakia (Syria)	CRIES-8; RCADS-25	High burden of PTSD symptoms and comorbid anxiety/depression in school-based sample; displacement and ongoing war-related stressors associated with symptoms.
Soykoek et al (2017) 6	Syrian children in a German refugee camp	Clinical/structured symptom assessment (correspondence)	Reports substantial PTSD symptom burden in camp setting; highlights need for psychosocial support and pediatric mental health services.
Kanan and Leão (2024) 7	Systematic review of youth exposed to Syrian conflict (multiple settings)	Varied	Synthesizes prevalence and determinants; emphasizes heterogeneity across measures and contexts and the need for higher quality longitudinal research.
Smeeth et al (2023) 3	Syrian refugee children/adolescents (6–18 years), informal settlements (Lebanon), longitudinal	PTSD symptom scales	Hair cortisol associated with earlier war exposure and current PTSD symptoms; patterns suggest HPA-axis alterations may vary by age and timing. 16
Soykoek et al (2019) 9	Syrian refugee children settled in Hatay (Turkey)	Standard symptom scales	Describes psychiatric symptom profiles in traumatized refugee children; comorbidity common; functioning influenced by social and family stressors.
Al-Smadi et al (2019) 1	Syrian adolescent refugees (Jordan)	Standard PTSD scales	PTSD symptoms present in adolescent refugee sample; risk influenced by cumulative exposure and post-migration stressors. 3
Alpak et al (2014) 12	Syrian refugees (Kurdistan region of Iraq), mixed age	PTSD and depression scales	Reports high burden of PTSD/depression; emphasizes contextual determinants and service gaps.
Katrinli et al (2022) 2	Mechanistic review (general PTSD)	N/A	Summarizes evidence that inflammation and immune dysregulation can accompany PTSD; directionality and causality remain uncertain.
Hazer and Gredebäck (2023) 13	Review focused on Syrian refugees and child development	N/A	Highlights cumulative pre-, peri-, and post-migration stressors affecting development; notes resilience and substantial evidence gaps.
Devakumar et al (2014) 8	Review of intergenerational effects of war (general)	N/A	Discusses pathways linking parental war exposure to child outcomes; intergenerational and epigenetic mechanisms are plausible but evidence remains evolving. 9
Felitti et al 10 /ACE review (2016)	Trauma-informed care review (general)	N/A	Frames early adversity as a driver of lifelong health risk and supports trauma-informed clinical approaches.
Theofanidis et al (2022) 14	Syrian refugees in Greece, adults	PTSD screening	Included for contextual comparison; adult findings underscore chronicity and comorbidity but are not pediatric-specific. 7

Abbreviations: HPA, hypothalamic–pituitary–adrenal; PTSD, posttraumatic stress disorder.

### Neuroendocrine Findings: HPA-Axis Alterations

Across pediatric trauma studies, HPA-axis findings in PTSD are heterogeneous. 6 Relative hypocortisol has been described, potentially reflecting differences in timing since trauma, chronicity, comorbid depression, pubertal stage, and measurement approach (e.g., saliva, plasma, diurnal slope, or hair cortisol). 12 In the Syrian context, longitudinal work using hair cortisol suggests that war exposure and PTSD symptoms can relate to altered cortisol output, with age and time since displacement potentially modifying associations. 8

### Immune and Inflammatory Findings

Mechanistic literature in PTSD supports possible elevations in pro-inflammatory markers such as IL-6, C-reactive protein (CRP), and tumor necrosis factor-alpha in some populations. 11 However, effect sizes and directions vary across studies, and whether inflammation precedes PTSD, results from PTSD, or reflects shared determinants (e.g., sleep disruption, infections, adiposity, poverty) remains unclear. 12 Pediatric conflict-specific data are limited; therefore, immune conclusions for Syrian children should be interpreted cautiously and viewed as biologically plausible hypotheses rather than established effects. 2 4 10 11

### Growth and Developmental Outcomes

Growth outcomes in conflict-affected children reflect overlapping risks: food insecurity, disrupted health care, recurrent infection, toxic stress, and reduced caregiving stability. Direct Syrian cohort studies linking PTSD specifically to anthropometric deficits are few; nonetheless, development-focused reviews highlight consistent associations between cumulative adversity and delays in cognitive, emotional, and educational functioning. Future Syrian pediatric studies should jointly measure mental health, nutrition, infection burden, and growth to separate PTSD-specific associations from correlated adversities. 3

### Family-Level and Intergenerational Pathways

Family functioning, caregiver mental health, and attachment security modify child outcomes after war exposure. 5 Intergenerational mechanisms, including stress-related epigenetic variation, have been proposed but should be considered preliminary. 6 Existing evidence supports the importance of caregiver well-being and stable caregiving environments as modifiable targets for intervention. 7

## Discussion

This targeted review supports three clinically relevant conclusions. First, Syrian conflict-affected children show a substantial burden of PTSD symptoms and comorbid internalizing problems. 12 Second, there is plausible biological embedding of trauma through stress and immune pathways, but pediatric conflict-specific evidence remains limited and heterogeneous, especially for immune markers and growth. 8 Third, family context and post-migration stressors are central determinants of risk and resilience and should be integrated into screening and intervention. 13

The main limitations are the observational nature of the evidence base, reliance on self-report symptom scales, variable trauma definitions, and limited Syrian cohort studies with biological measures. 8 We therefore avoid precise quantitative claims across domains and emphasize patterns and uncertainties. 10 Future work should prioritize prospective designs, prespecified analyses, careful control of nutrition and infection confounding, and transparent reporting of assays and sampling protocols.

## Conclusions

War-related PTSD in children is associated with broad risks to mental health and may coincide with measurable alterations in stress biology. For Syrian children, evidence is strongest for high mental health burden and emerging for HPA-axis changes; immune and growth impacts remain less directly established. Trauma-informed, family-centered interventions and longitudinal research are priorities to reduce long-term harm.
